# Postoperative Stroke Through a Patent Foramen Ovale in a Patient With Lung Cancer: A Case Report

**DOI:** 10.7759/cureus.89618

**Published:** 2025-08-08

**Authors:** Masaya Nakamura, Yuki Shimizu, Tatsuya Goto, Terumoto Koike, Masanori Tsuchida

**Affiliations:** 1 Division of Thoracic and Cardiovascular Surgery, Niigata University Graduate School of Medical and Dental Sciences, Niigata, JPN

**Keywords:** cerebral infarction, deep vein thrombosis, lung cancer surgery, paradoxical brain embolism, patent foramen ovale, postoperative stroke, pulmonary resection, thoracic surgery complication

## Abstract

Cerebral infarction is a rare but serious complication after pulmonary resection for lung cancer. A 78-year-old man with hypertension and diabetes underwent video-assisted thoracoscopic right middle lobectomy for stage IA2 adenocarcinoma. On postoperative day 1, he developed acute right hemiparesis and motor aphasia. Diffusion-weighted magnetic resonance imaging revealed acute infarction in the left frontal lobe. Cerebral angiography showed no steno-occlusive lesions in the major cerebral arteries but demonstrated distal peripheral artery occlusions. Evaluation, including Holter monitoring, chest computed tomography, and venous ultrasound, identified no atrial fibrillation or other common embolic sources, but transthoracic echocardiography with an agitated-saline test confirmed a patent foramen ovale (PFO), suggesting paradoxical brain embolism (PBE) as the likely etiology. Treatment was initiated with aspirin on postoperative day 2, switched to apixaban after PFO confirmation. The patient was transferred to a rehabilitation facility on postoperative day 23, with marked improvement in paresis and aphasia, returning to near-baseline activities of daily living. Perioperative strokes are often observed in patients with multiple underlying risk factors. In pulmonary resection, pulmonary vein stump thrombosis has been proposed as a causative mechanism, although PBE through PFO warrants consideration as an important alternative etiology because lung resection creates ideal conditions for PBE by combining increased venous thrombosis risk with elevated right heart pressures due to loss of the pulmonary vascular bed.

## Introduction

Cerebral infarction following lung cancer surgery is a rare complication with serious adverse effects and occurs in approximately 0.9% of cases [1,2]. Generally, perioperative strokes are observed in patients with multiple underlying risk factors, including advanced age, atrial fibrillation, diabetes mellitus, and chronic kidney disease [3]. A patent foramen ovale (PFO) is a congenital condition where a small opening in the atrial septum, part of fetal circulation, persists after birth, and is present in about 25% of the general population, significantly linked to an increased risk of ischemic stroke [4-7]. In pulmonary resection, thrombus formation at a resected pulmonary vein stump has been proposed as an etiology of periprocedural embolic stroke, with subsequent embolization into cerebral circulation [2,8]. However, the altered hemodynamics following pulmonary resection, such as increased right heart pressure due to loss of the pulmonary vascular bed, may also facilitate paradoxical brain embolism (PBE) through a PFO [9,10]. Herein, we present a case that illustrates the potential role of PBE through an unrecognized PFO as a consideration in the differential diagnosis of postoperative stroke in patients undergoing lung resection.

## Case presentation

A 78-year-old man was referred to our hospital for surgical management of lung cancer. He is a known hypertensive and diabetic with a history of surgical resection for renal pelvic cancer. He had a smoking history of 118 pack-years. His preoperative findings are summarized in Table 1.

**Table 1:** Baseline preoperative laboratory values, tumor marker levels, pulmonary function parameters, and electrocardiogram findings CBC, complete blood count; WBC, white blood cell count; CRP, C-reactive protein; eGFR, estimated glomerular filtration rate; CEA, carcinoembryonic antigen; SCC, squamous cell carcinoma antigen; VC, vital capacity; FEV_1_, forced expiratory volume in 1 second; FVC, forced vital capacity; ECG, electrocardiogram

Parameter	Result	Reference range
CBC		
WBC (×10³/µL)	6.33	3.3–8.6
RBC (×10⁶/µL)	4.32	4.3–5.5
Hemoglobin (g/dL)	13.9	13.7–16.8
Platelets (×10³/µL)	210	158–348
Serum chemistry		
Total protein (g/dL)	7.6	6.6–8.1
Albumin (g/dL)	4.2	4.1–5.1
CRP (mg/dL)	0.1	< 0.14
Renal function		
Creatinine (mg/dL)	1.31	0.6–1.1
eGFR (mL/min/1.73m²)	41.5	> 90
Tumor markers		
CEA (ng/mL)	9.4	< 5.9
SCC antigen (ng/mL)	2.2	< 1.5
Pulmonary function		
VC (L, % predicted)	3.45 (102.9%)	
FEV₁ (L)	2.42	
FEV₁/FVC (%)	71.6	
ECG	Normal sinus rhythm, 57 bpm	

**Figure 1:**
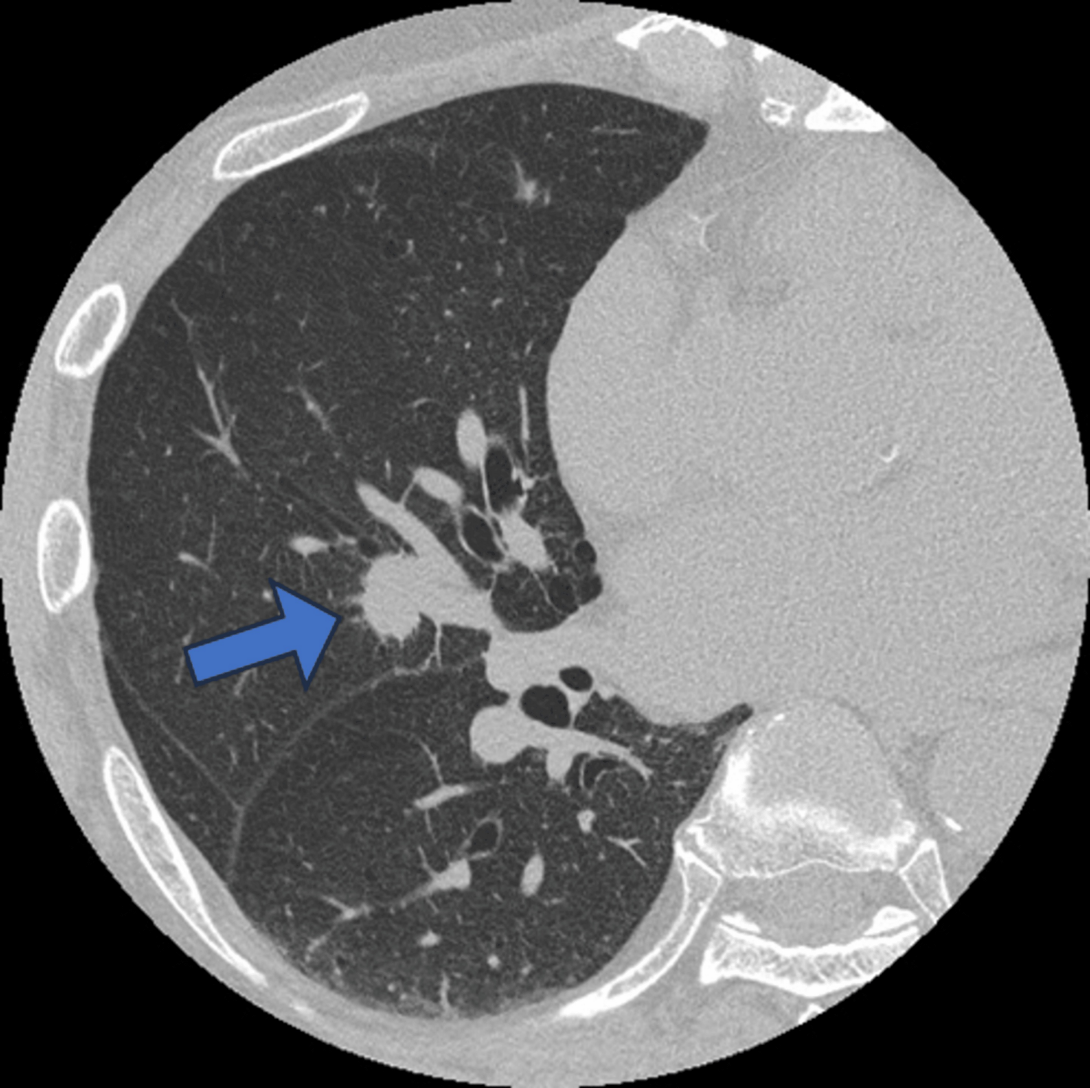
Preoperative chest computed tomography The axial image shows a solid nodule in the right middle lobe of the lung (arrow). This lesion was confirmed to be an adenocarcinoma using bronchoscopic biopsy.

The patient was diagnosed with adenocarcinoma of the right middle lobe of the lung (Figure 1), which was confirmed by bronchoscopic biopsy and staged as cT1bN0M0, stage IA2 (Union for International Cancer Control/American Joint Committee on Cancer, 8th edition) [11]. He subsequently underwent a video-assisted thoracoscopic right middle lobectomy with ND2a-2 lymph node dissection. Operative time was 2 h 42 min, and estimated blood loss was 40 mL.

**Figure 2:**
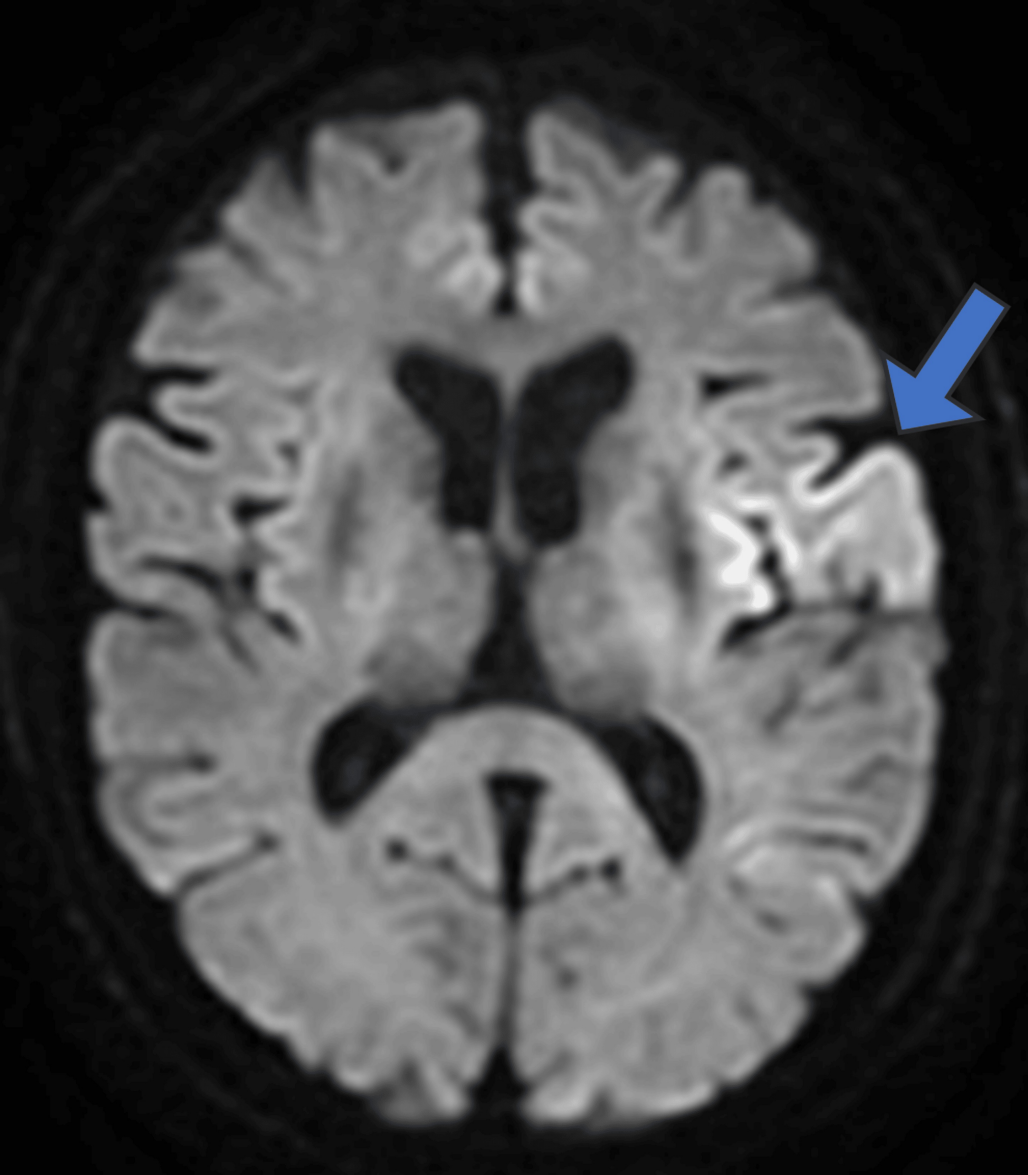
Brain magnetic resonance imaging on postoperative day 1 An axial view of diffusion-weighted imaging demonstrating a high signal intensity in the left frontal lobe. The lesion spans the territories of both the anterior and the middle cerebral arteries, of which findings are consistent with an acute cerebral infarction (arrow).

Mobilization began on postoperative day (POD) 1. On the afternoon of POD 1, while escorting visitors, the patient suddenly collapsed and developed incomplete right hemiparesis with motor aphasia. A non-contrast head computed tomography (CT) showed no hemorrhage, whereas diffusion-weighted magnetic resonance imaging revealed high signal intensity in the left frontal lobe within both the anterior and middle cerebral artery territories (Figure 2), which was consistent with acute infarction. Subsequent cerebral angiography showed no steno-occlusive lesions in the major cerebral arteries, thereby ruling out a high suspicion of atherothrombotic etiology. However, distal occlusions were noted in the peripheral cerebral arteries, and mechanical thrombectomy was not indicated because there were no steno-occlusive lesions in the major cerebral arteries.

**Figure 3:**
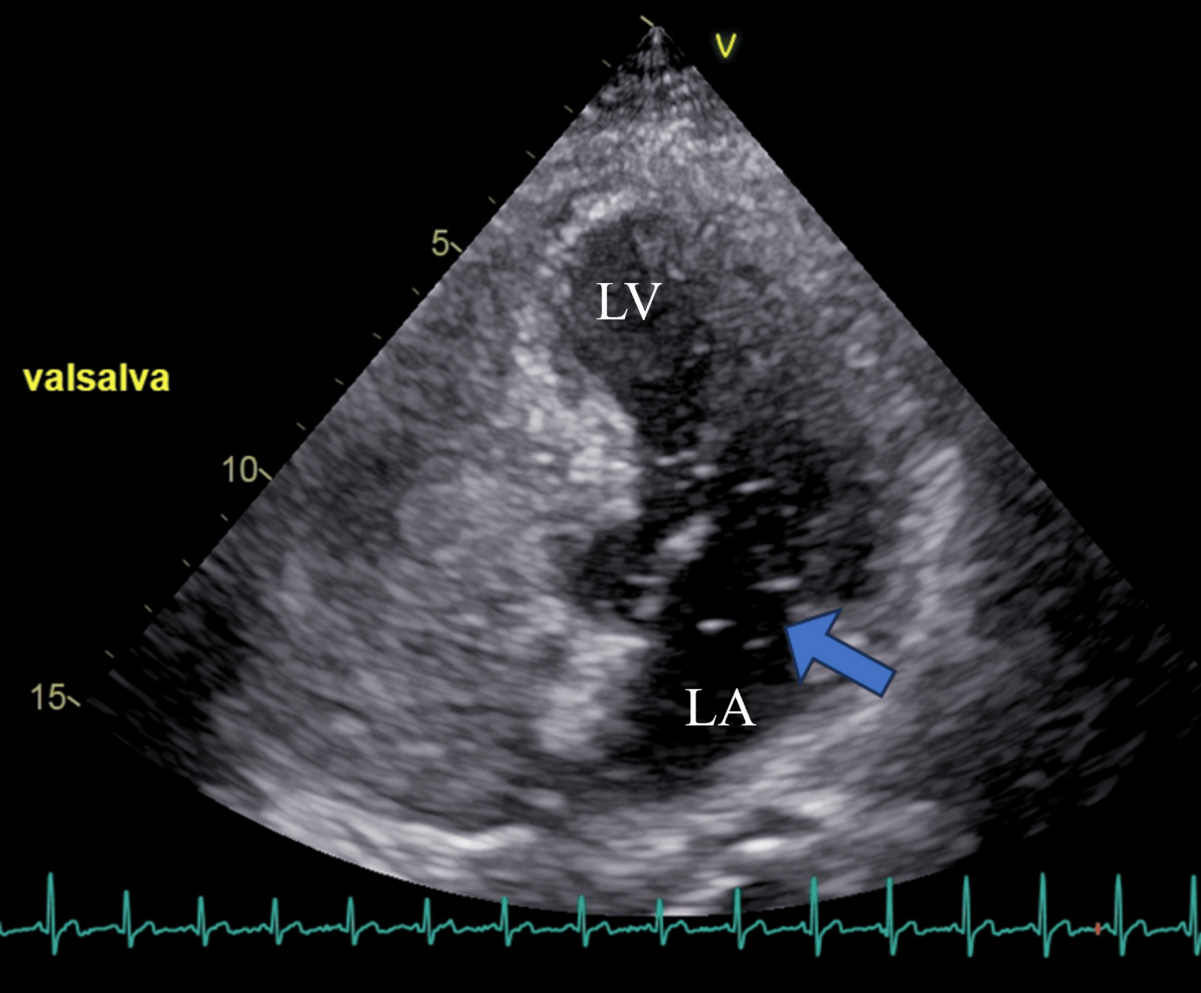
Patent foramen ovale confirmed using an agitated-saline (bubble) test Following a Valsalva maneuver, microbubbles (arrows) are seen passing from the right atrium into the left atrium (LA) and left ventricle (LV), confirming a right-to-left shunt through a patent foramen ovale.

The stroke was preliminarily categorized as an embolic stroke of undetermined source, and aspirin was initiated on POD 2. Evaluation to identify the embolic source included 24-h Holter monitoring on POD 6, which showed no arrhythmia; contrast-enhanced chest CT on POD 12, showing no cardiac or pulmonary vein stump thrombus (PVST); lower extremity venous ultrasound on POD 5, detecting no deep vein thrombosis (DVT); and transthoracic echocardiography (TTE) with an agitated-saline (bubble) test on POD 8, confirming a PFO with microbubbles appearing in the left atrium and left ventricle within three cardiac cycles during a Valsalva maneuver (Figure 3). Carotid imaging was not performed as cerebral angiography had ruled out major steno-occlusive lesions. Although no venous thrombus was identified, the clinical course was highly suggestive of PBE; therefore, aspirin was switched to apixaban. The patient was transferred to a rehabilitation facility on POD 23. With rehabilitation, the right-sided paresis and aphasia markedly improved, and he returned to nearly his preoperative level of activities of daily living. Follow-up at 10 months post-surgery showed no recurrence of lung cancer or stroke.

## Discussion

In the context of pulmonary resection, since the report by Ohtaka et al. in 2013 linked cerebral infarction to PVST after left upper lobectomy, embolism from a stump thrombus has been considered a significant cause of postoperative stroke [8]. However, Hattori et al. found that although the incidence of PVST was higher on the left side, there was no significant difference in the incidence of cerebral infarction regardless of its presence [2]. This finding suggests that postoperative strokes after pulmonary resection may result from a combination of general perioperative risk factors and procedure-specific mechanisms, including PVST and PBE through PFO.

A PFO is a condition in which a small communication persists into adult life because the foramen ovale, a normal component of the fetal circulation, fails to close after birth through the fusion of the two embryologic components of the atrial septum, the septum primum and septum secundum [4]. Although left atrial pressure is typically higher than that in the right, a PFO can act as a one-way valve, allowing a transient right-to-left shunt when right atrial pressure increases, such as during coughing or a Valsalva maneuver [4]. This shunt is rarely observed at rest; therefore, diagnosis often requires provocative testing, such as contrast-enhanced TTE with a Valsalva maneuver, using an agitated saline solution as the contrast material [4]. PFO is present in about 25% of the general population and is by no means rare, highlighting its potential relevance in postoperative stroke evaluation [5,6].

PBE occurs when a venous thrombus, often originating from a DVT, passes through a PFO and enters the systemic arterial circulation, causing a stroke [12]. According to the diagnostic criteria proposed by Ueno et al., “Definite PBE” requires the presence of a PFO along with (1) imaging evidence of an embolic stroke; (2) DVT or pulmonary embolism; and (3) the absence of other embolic sources [12]. This definite subgroup accounted for 5% of all acute cerebral infarctions in their series [11]. A case fulfilling two of these three criteria is classified as “Probable PBE” [12]. Ueno et al. noted that some cases classified as “Probable” should have actually been included in the “Definite” group [12]. The present case, with no confirmed DVT, is therefore classified as “Probable PBE.” Given that the patient underwent a middle lobectomy (a procedure with a relatively low risk of PVST) and had a confirmed PFO, PBE appears to be the most reasonable etiology [2,12]. The successful diagnosis of PBE through a comprehensive evaluation, including TTE, underscores the importance of routine thorough assessment in postoperative settings. The switch from aspirin to apixaban was made to reduce the risk of recurrent thromboembolism associated with the confirmed PFO.

Our institutional protocol for DVT prophylaxis in patients without pre-existing anticoagulant use, such as the present case, comprises compression stockings and an intermittent pneumatic compression (IPC) device. In this case, IPC was removed on the morning of POD 1, while compression stockings remained in use at the time of stroke onset in the afternoon. IPC is a well-documented strategy for significantly reducing the risk of DVT in lung cancer surgery [13]. Despite the application of such standard prophylaxis, the risk of DVT cannot be completely eliminated, and its prevention remains a clinical challenge [13].

PBE has historically been regarded as a condition primarily affecting younger individuals [14,15]. However, recent data have demonstrated a strong association between PFO and PBE, even in older adults [6]. This is attributed to a higher rate of DVT and hemodynamic changes, such as increased pulmonary artery pressure, which facilitate transient right-to-left shunt [16,17]. Additionally, a primary hemodynamic consequence of lung resection is an increase in right ventricular afterload due to loss of the pulmonary vascular bed [10].

Okada et al. reported that in the early postoperative period following lung resection, the right ventricular end-diastolic volume index at rest gradually increased, showed a statistically significant elevation compared with the preoperative value on POD 2, and returned to baseline by the third postoperative week [9]. Thus, in a patient with PFO, the perioperative period of lung resection combines the two fundamental requirements for PBE: an increased risk of venous thrombosis and the specific hemodynamic trigger-elevated right atrial pressure-that facilitates a right-to-left shunt [9,13].

PFOs associated with PBE carry a risk of recurrence, and catheter-based closure is generally indicated in patients aged under 60 years [18-21]. Given the patient's age of 78 years, PFO closure was not considered. Furthermore, if the right-to-left shunt is caused by a temporary increase in right heart pressure following lung resection, the necessity of treating the PFO itself remains unclear. It is also known that PFO closure carries a risk of inducing atrial fibrillation (Afib) and is not typically indicated for patients with existing Afib, who likely need anticoagulation regardless of arrhythmia risk [18-21].

## Conclusions

We have reported a case of cerebral infarction following a right middle lobectomy in which PBE due to a previously unrecognized PFO was strongly suggested as the cause. In addition to PVST, PBE through a PFO represents an important differential diagnosis for postoperative stroke in patients after lung resection. This case suggests that clinicians should consider PBE when evaluating postoperative stroke after lung resection.
